# Tissue resolved, gene structure refined equine transcriptome

**DOI:** 10.1186/s12864-016-3451-2

**Published:** 2017-01-20

**Authors:** T. A. Mansour, E. Y. Scott, C. J. Finno, R. R. Bellone, M. J. Mienaltowski, M. C. Penedo, P. J. Ross, S. J. Valberg, J. D. Murray, C. T. Brown

**Affiliations:** 1 Department of Population Health and Reproduction, University of California, Davis, Davis, USA; 2 Department of Clinical Pathology, College of Medicine, Mansoura University, Egypt, Mansoura, Egypt; 3 Department of Animal Science, University of California, Davis, Davis, USA; 4 Veterinary Genetics Laboratory, University of California, Davis, Davis, USA; 5 Large Animal Clinical Sciences, Michigan State University, College of Veterinary Medicine, East Lansing, USA

**Keywords:** Equine transcriptome, Tissue-specificity, RNA-seq

## Abstract

**Background:**

Transcriptome interpretation relies on a good-quality reference transcriptome for accurate quantification of gene expression as well as functional analysis of genetic variants. The current annotation of the horse genome lacks the specificity and sensitivity necessary to assess gene expression especially at the isoform level, and suffers from insufficient annotation of untranslated regions (UTR) usage. We built an annotation pipeline for horse and used it to integrate 1.9 billion reads from multiple RNA-seq data sets into a new refined transcriptome.

**Results:**

This equine transcriptome integrates eight different tissues from 59 individuals and improves gene structure and isoform resolution, while providing considerable tissue-specific information. We utilized four levels of transcript filtration in our pipeline, aimed at producing several transcriptome versions that are suitable for different downstream analyses. Our most refined transcriptome includes 36,876 genes and 76,125 isoforms, with 6474 candidate transcriptional loci novel to the equine transcriptome.

**Conclusions:**

We have employed a variety of descriptive statistics and figures that demonstrate the quality and content of the transcriptome. The equine transcriptomes that are provided by this pipeline show the best tissue-specific resolution of any equine transcriptome to date and are flexible for several downstream analyses. We encourage the integration of further equine transcriptomes with our annotation pipeline to continue and improve the equine transcriptome.

**Electronic supplementary material:**

The online version of this article (doi:10.1186/s12864-016-3451-2) contains supplementary material, which is available to authorized users.

## Background

Transcriptomics is rapidly evolving from a focus on novel gene identification to resolving structural gene details. The transcriptomes of better-studied organisms, such as Drosophila, mouse and human have been updated to accommodate for this transition [1–3]. However, for less well characterized animals, such as the horse, there is often only annotation of a single variant of a gene and with insufficient annotation of multiple splice variants, UTR extensions and non-protein coding RNA. This lack of information can challenge subsequent differential gene expression analyses and functional studies. There have been several attempts to improve the equine transcriptome with single tissue transcriptomes from lamellar tissue [4] or peripheral blood mononuclear cells [5] and from pooled composites of various tissues [6, 7], however a broader effort defining and integrating many tissue-specific transcriptomes and obtaining the library depth and strand information required to capture gene complexity is still needed.

ENSEMBL and NCBI provide publically available annotations for several vertebrate genomes including horse [8]. Both underlying annotation pipelines integrate homology search and ab initio prediction; however accurate UTR prediction and isoform recognition require species-specific transcriptional evidence [9, 10]. For this equine transcriptome, the transcriptional evidence provided by total RNA sequencing (RNA-seq) was the basis of our gene annotation. This approach permits more reliable discovery of novel genes and isoforms, extension of UTRs and the flexibility necessary to establish a balance between sensitivity and specificity of gene detection for downstream applications.

Our annotation integrates the benefits of increased depth in reads and strand-specificity, for some tissues, as well as using a range of tissues from many horses, which allows tissue-specific transcriptomes to be extracted. We have incorporated RNA-seq from a diverse set of 8 tissues ranging from the central nervous system (CNS), skin and skeletal muscle tissues in adults to the inner cell mass (ICM) and trophectoderm (TE) in embryonic tissues (Table 1). The diversity in age, sex and tissue of the samples included in our assembly supply the equine transcriptome with its best spatiotemporal resolution and most complete gene UTR definition to date.

**Table 1:** Sample and library preparations used as input for our equine transcriptome

Tissue	Library Preparation	Library Characteristics	#Samples	#Frag (M)	#bp (Gb)	Reference
Brainstem	RiboRNA-depleted	PEl00bp, stranded	8*	166.73	33.68	Finno et al., 2016 [13]
Cerebellum	RiboRNA-depleted	PEl00bp, stranded	12	411.48	82.3	Scott et al., 2016 [23]
Muscle	Poly-A capture	PE125bp, stranded	12	301.94	76.08	
Retina	Poly-A captured	PE80bp unstranded	2	20.3	3.28	Bellone et al., 2013
Spinal Cord	RiboRNA-depleted	PEl00bp, stranded	16*	403	81.4	Finno et al., 2016 [13]
Skin	Poly-A captured	PE80bp, unstranded	2	18.54	3	Holl et al., 2016
	Poly-A captured	SE80bp, unstranded	2	16.57	1.34	Holl et al., 2016
	Poly-A captured	SE95bp unstranded	3	105.51	10.02	Bellone et al., 2013
Embryo ICM	Ovation RNA-seq	PEl00bp, unstranded	3	126.32	25.26	Iqbal et al., 2014
	Ovation RNA-seq	SEl00bp, unstranded	3	115.21	11.52	Iqbal et al., 2014
Embryo TE	Ovation RNA-seq	PEl00bp, unstranded	3	129.84	25.96	Iqbal et al., 2014
	Ovation RNA-seq	SEl00bp, unstranded	3	102.26	10.23	Iqbal et al., 2014
Total				1917.7	364.07	

Notes: *Seven individuals had both brainstem and spinal cord tissue collected from them. Seven of the skin samples were taken from 5 individuals and one individual had both retina and skin sampled, bringing our total number of individuals to 59

We recognize that availability of annotation criteria and integration of transcriptome data is paramount for systematically improving the equine transcriptome. Our goal is to encourage equine researchers to incorporate their transcriptomic data using our pipeline as the common annotation pipeline and our initial transcriptomes as a reference framework. We intend to continue improving equine gene annotation through better UTR definition, isoform splicing characterization and novel gene identification. The annotation presented in this paper will improve the gene structure definition in current databases and the accuracy of downstream analyses, including both differential gene expression analysis and genetic variants annotation in the horse.

## Results

### Overall mapping statistics and gene counts after filtration

**Fig. 1:**
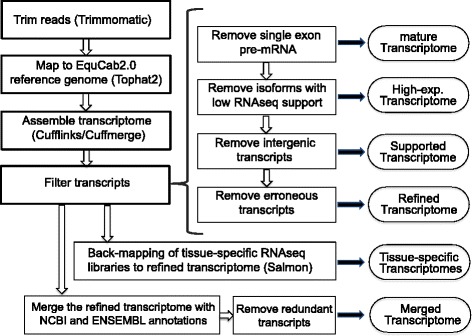
An outline of the workflow used to generate each version of the transcriptome. Transcriptome products are in *ovals*. Programs used to perform various steps are indicated in *parentheses*. All transcriptome versions and the pipeline scripts are publically available

RNA-seq of 59 samples in 12 libraries from 8 different horse tissues provided 1917.7 million fragments and 364 Gb of sequence bases. A summary of the library preparation, number of horses per library and total number of fragments and bases provided by each tissue library can be found in Table 1. The overall average mapping rate for Tophat2 was ~83% with concordance rates ranging from 29 to 89% (average 75%) for paired end libraries. Concordance rates seem to be affected by the type of library preparation, where polyA selected and strand-specific libraries have the best rates. Library specific mapping rates can be found in Additional file 1: Table S1. The initial Cufflinks assembly identified 117,019 genes/211,562 transcripts. After this initial analysis we applied four grades of filtration (Fig. 1). Primary filtration of transcripts removed the likely pre-mRNA fragments by eliminating single exon transcripts that were present within introns or overlapping with exons of other multi-exon transcripts. After primary filtration there were 75,102 genes/162,261 transcripts. The second filter was implemented to remove isoforms likely to be experimental artifacts by excluding low abundant transcripts with less than 5% of total expression for their locus. The remaining 114,830 transcripts represented 75,375 genes. In the third filter, non-coding transcripts that lack any supporting evidence from NCBI or ENSEMBL annotations, non-horse gene models (“Other RefSeq” and “TransMap RefGene” UCSC tracks) or ab initio predictions (“Augustus”, “Geneid”, “Genscan” and “N-SCAN” UCSC gene prediction tracks) were excluded. This third filtered version of the transcriptome has 76,323 transcripts in 37,062 genes. The last filter was for removing likely erroneous transcripts. The mtDNA in mammals is known for gene overlapping and polycistronic expression [11], permitting inaccurate prediction of mitochondrial transcripts by Cufflinks; we therefore excluded the mitochondrial contigs from our filtered assembly. Also, short transcripts less than 201 bp (192 transcripts in 184 genes) were removed because they are more likely to represent repetitive sequences or incomplete gene fragments. Once erroneous transcripts were removed, our final refined version of the transcriptome contained 36,876 genes (76,125 transcripts) including 15,343 single exon transcripts, 8808 two-exon transcripts, and 51,974 transcripts with three or more exons. A version of our refined transcriptome that is merged with the NCBI and ENSEMBL annotations, with redundant transcripts removed, is also available. This is the most comprehensive product of our pipeline and is valuable for differential gene expression analysis in tissues other than those provided in our assembly. Summary statistics including N50, number of genes, total size and average length of fragment for all six versions of the transcriptome can be found in Additional file 2: Table S2.

### Comparison between our transcriptome and currently available equine transcriptomes

**Fig. 2:**
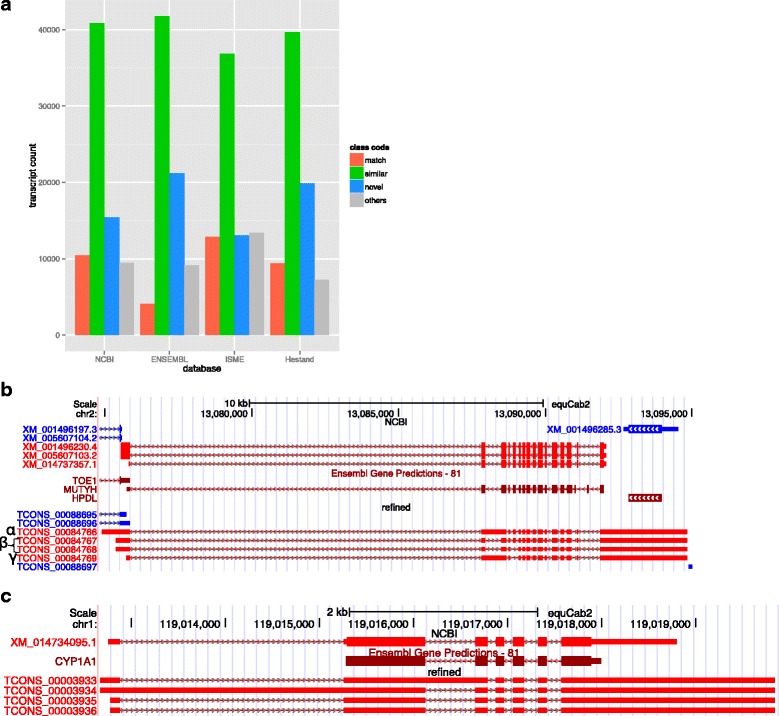
Comparison of our refined transcriptome to current equine annotations. The degree of similarity between our refined transcriptome and current annotations can be found in (**a**). The annotation of *MUTYH* in the refined version of the transcriptome shows the addition of several isoforms, α, β, and γ, as seen in the human, of *MUTYH* (**b**). The gene annotation of *CYP7A1* in the refined transcriptome also shows the inclusion of an extended alternative first exon not seen in other species (**c**)

We performed a comparison between our transcriptome and gene models from NCBI, ENSEMBL and two published equine transcriptomes, that we refer to as Hestand [7] and ISME [5] (Table 2 and Additional file 3: Table S3). In our comparisons, transcripts sharing one or more splice junctions are considered similar but only those with identical intron chains are matching. The comparison reveals that the matching transcripts between our refined transcriptome and NCBI annotation are greater than 2.5-fold those matching the ENSEMBL annotation. However the highest number of matching transcripts occurred with the ISME transcriptome with 12,849 transcripts (Fig. 2a). About 50% of the refined transcripts have a similar match in all the public transcriptomes. Evidence of improvements to the annotation of genes with a similar match to other assemblies can be found in genes such as *MUTYH*, where the three major isoforms annotated in humans [12] are now distinguishable in the horse (Fig. 2b). The gene *CYP7A1* is another example where a novel first exon has been annotated and extended in our version of the transcriptome [13] (Fig. 2c). About 20 and 28% of the refined transcripts are novel when compared to NCBI and ENSEMBL annotations respectively. Combined, there are 22,641 transcripts in candidate novel loci. Our approach of applying four successive steps of filtration strictly qualifies our novel isoforms as transcripts with ORFs or exonic overlap with candidate gene models. Mainly, novel transcripts contained within introns of other genes were excluded to avoid the artifacts of retained intronic reads, common in rRNA depleted libraries. Using the NCBI model as a reference for comparison, our novel transcripts from the refined transcriptome have no bias towards any particular chromosome after accounting for chromosome size (Additional file 4: Figure S1). In order to calculate the gene and isoform detectability of our transcriptome compared to current annotation, we calculated sensitivity and specificity [14] between our transcriptome and a reference and found that, using NCBI as the reference, our transcriptome had a 78.8% sensitivity and 23.8% specificity at the base level and a 32% sensitivity and 21.1% specificity at the locus level. Detailed pairwise assessment for all equine annotations can be found in Additional file 5: Table S4. We developed a statistic to assess the conflict between different assemblies, termed “complex loci”, which refer to the loci that represent one gene locus in one transcriptome and two or more gene loci in another. Our transcriptome has 1355 and 997 transcripts that were considered complex loci between our transcriptome and NCBI and ENSEMBL, respectively. The Hestand transcriptome, however, has less with 660 and 798 complex loci against the NCBI and ENSEMBL, respectively. The ISME transcriptome has substantially more, with 1546 and 1226 complex loci when compared to NCBI and ENSEMBL, respectively.

**Table 2:**
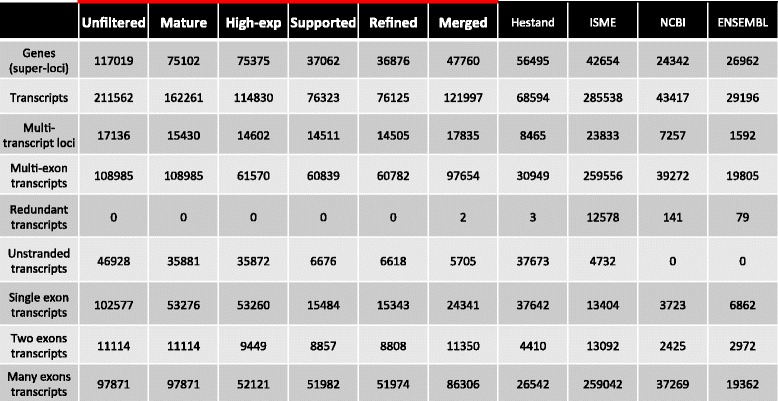
Comparison of current public equine annotations to six versions of our transcriptome (bolded and outline in red) in terms of gene numbers and composition

### UTR extension

To test the effect of the new assembly on the UTRs of known genes, we identified the protein coding isoforms sharing the exact intron chain with NCBI isoforms, which yielded 9736 isoforms from 7419 genes. The difference in the total length of each transcript was then calculated and we found that we extended the length of 8899 isoforms (6817 genes) by 29.7 Mb in total. 831 isoforms (718 genes) lost 0.3 Mb in total with an average of 0.4 kb per isoform, while 6 isoforms did not change.

### Gene and isoform distinctions between tissue-specific transcriptomes

**Fig. 3:**
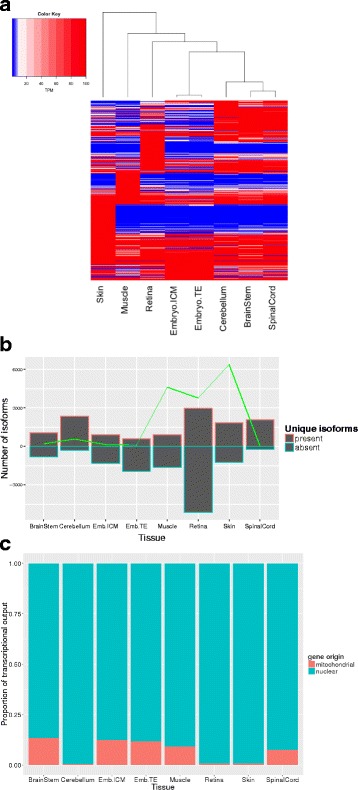
Tissue-specific gene and isoform composition of the transcriptome. A heatmap of genes with high expression and substantial expression differences across tissues (**a**). A bar graph showing isoforms uniquely present (the bar outlined in *red* above the x-axis) or solely absent (the *blue* outlined bars extending below the x-axis). The *green trendline* corresponds to the cumulative TPM of the uniquely present transcripts (**b**). A stacked bar graph showing the transcription percentage of mitochondrial genes versus nuclear encoded genes (**c**). Emb. Is short for embryo

We selected genes with high expression (a sum of TPMs across all tissues above 200) and substantial expression differences across tissues (a standard deviation above 200). Unsupervised hierarchical clustering grouped genes that may be co-expressed as well as illustrating the relationship between the tissue-specific transcriptomes. As expected, the transcriptomes from the three central nervous system (CNS) tissues clustered together, as did the two embryonic tissues, with the skin and skeletal muscle furthest from these clusters (Fig. 3a). Blocks of genes showing uniquely high expression in a given tissue were further annotated with NCBI gene names and then summarized with Panther biological processes annotations. The top two Panther pathways (lowest *p*-values) for each of these gene blocks are reported in the text below, with the full Panther annotation tables detailed in Additional file 6: Table S5. The CNS cluster contained overrepresented processes regarding brain function and development: nervous system development (*p* = 2.10E-6, fold-enrichment = 7.12) and neurogenesis (*p* = 9.36E-5, fold-enrichment = 7.73). The retina contained processes consisting of photoreception and visual perception: phototransduction (*p* = 3.52E-08, fold-enrichment = 80.75), and visual perception (*p* = 3.69E-18, fold-enrichment = 37.64). The skeletal muscle encompassed genes pertaining to muscle physiology and regulation: muscle contraction (*p* = 1.48E-27, fold-enrichment =57.58) and myofibril assembly (*p* = 6.05E-11, fold-enrichment = 72.8). The embryonic tissues have the most general processes assigned to their distinct clusters: translation (*p* = 1.15E-11, fold-enrichment = 16.35) and peptide biosynthetic processes (*p* = 1.95E-11, fold-enrichment = 15.8). And finally, the skin consisted of processes concerning epithelial organization and production: intermediate filament organization (*p* = 1.69E-07, fold-enrichment > 100) and skin development (*p* = 1.89E-09, fold-enrichment = 22.49).

When attention is given to the isoforms showing unique presence or sole absence in a tissue, the cerebellum and retina possess the most isoforms that are uniquely present, with the retina also containing the largest amount of solely absent isoforms (Fig. 3b). The uniquely present transcripts in the retina have Panther annotations of visual perception and photoreceptor cell differentiation, and in the cerebellum they have annotations for nervous system development and generation of xneurons. The transcripts solely absent from the retina pertain mainly to positive regulation of DNA replication (*p* = 2.49E-03, fold-enrichment = 3.53) and anatomical structure development (*p* = 2.72E-19, fold-enrichment = 1.48) (Additional files 7 and 8: Table S6 and S7). Utility of these isoforms, in terms of expression, is strongest in the skin, retina, skeletal muscle and to a small extent the cerebellum (Fig. 3b). Despite these differences in unique isoforms, multi-exons transcripts and multi-transcript loci, the splicing rate across tissues, as calculated by Cuffcompare [15], ranges from 1.7 to 1.9 (Table 3).

**Table 3:** Tissue-specific splicing rate as calculated by Cuffcompare, with relevant number of multi-exonic transcripts and multi-transcript loci per tissue

	Embryo ICM	Embryo TE	Skin	Brainstem	Cerebellum	Retina	Spinal cord	Muscle
Genes	33,998	32,050	30,003	34,792	36,139	26,733	34,980	29,549
Transcripts	57,400	54,424	51,995	62,993	66,364	47,095	66,001	52,000
multi-exon transcripts	44,069	42,433	42,432	49,346	51,640	39,420	52,175	42,483
Multi-transcript loci	11,938	11,461	11,797	13,066	13,334	10,866	13,352	11,560
Splicing rate	1.7	1.7	1.7	1.8	1.8	1.8	1.9	1.8

Nuclear coding versus mitochondrial encoded genes were parsed out per tissue to determine how much of the sequencing resources are allocated to genes of the mitochondria (Fig. 3c), with the conclusion that the brainstem, spinal cord, embryonic tissues and skeletal muscle exhibit the largest proportions of transcriptional output devoted to mitochondrial genes.

### Classification and annotation of novel genes

**Fig. 4:**
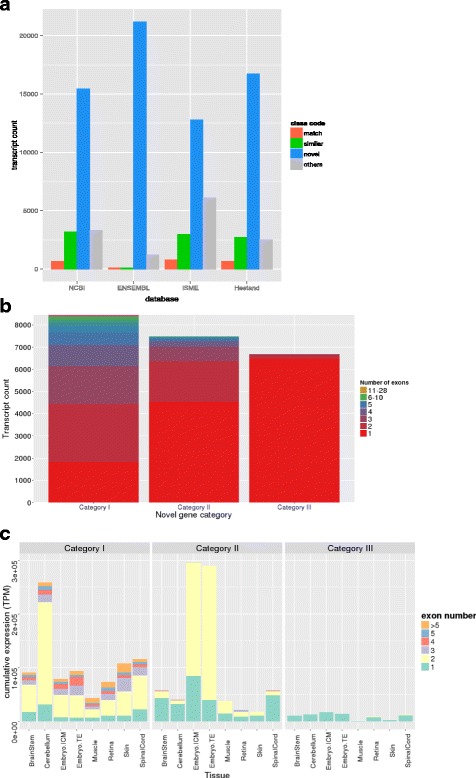
Novel gene analysis and classification. A bar graph showing the comparison of all the novel genes against the current equine annotations (**a**). The three categories of novel genes were supported novel genes (Category I), unsupported, but conserved, novel genes (Category II) and the unsupported, un-conserved, but novel genes with an ORF (Category III). A stacked bar graph of transcript counts with all three categories of novel genes showing exonic composition (**b**) and their cumulative TPM in a tissue specific manner (**c**)

In total there were 22,640 novel transcripts, with varying levels of support from current equine annotations (Fig. 4a). Classification of our novel genes was necessary to better represent how our transcriptome contributed to novel gene identification. Three categories of novel genes based upon the supportive evidence within and across species were made with each successive category being less supported by equine or orthologous gene models. Our first category of novel genes hosts those missing from NCBI and/or ENSEMBL annotation, but supported by either NCBI, ENSEMBLE, Hestand or ISME annotations (Category I). The second group of novel genes were novel to all public equine annotations, but conserved by means of orthologous gene similarity or supported by possible gene prediction (Category II). The third category of novel genes were unsupported by any candidate gene models, but had an ORF (Category III). Category I has a total of 8459 transcripts, with 2/3 of these transcripts absent from the ENSEMBL annotation (Additional file 9: Figure S2). Another 1849 transcripts in this category are novel to both NCBI and ENSEMBL annotations, yet supported by Hestand or ISME annotations. Homology with the SWISS-prot database identified at least one significant (*p* < 1E-10) hit for almost half the transcripts in this Category I (Additional file 10: Table S8). The second category has 7494 transcripts that – unless on the opposite strand - do not overlap with known gene models in public annotations. Annotation of these transcripts was performed partially by testing overlap with non-horse gene models and also by homology search. Only 16% of these transcripts have significant hits against the SWISS-prot database (Additional file 10: Table S8). The third category of novel genes includes 6687 transcripts with an ORF as the only functional support for these transcripts. The first category of novel genes shows the most diverse distribution of exon numbers comprising the genes (ranging from 1 to 28), whereas the unsupported genes contained mainly single exon genes (Fig. 4b). The expression analysis of the three novel gene categories shows a clear reduction of cumulative expression from category I to III.  There was also an obvious tissue-specific pattern in the expression of novel genes.  Supported novel genes (Category I) had the highest expression in the cerebellum and spinal cord, which consisted mainly of genes with up to three exons. However, when looking at only the second category of novel genes, the embryo contributed the highest expression of novel transcripts, which mainly consisted of transcripts with two exons. Category III novel transcripts mainly consisted of single exon transcripts and showed similarly low expression across all tissues (Fig. 4c).

## Discussion

Using RNA-seq from 59 horses across eight tissues has allowed us to capture transcriptome complexity and provide spatial resolution in terms of tissue-specificity in manner that exceeds any current equine annotations. Our descriptive statistics and accessible pipeline make this project open to modifications and further integration of transcriptomes.

RNA-seq based transcriptomes are prone to false inflation of gene numbers for several reasons. Technical limitations such as limited sequencing read length, amplification errors, false splicing events, and assembler deficiencies are among several reasons causing misassembly. Pervasive transcription is another predominant source of such inflation [16–18]. Some types of sequencing libraries increase the problem as well; for example rRNA depletion inflates the assembly with primary transcripts and false isoforms exhibiting intronic retention [19]. Our pipeline takes these factors into account and runs unguided by a reference transcriptome with several transcript filtration steps aimed to reduce inclusion of inaccurate transcripts, while retaining the sensitivity for novel transcript detection. The effect of this procedure can be seen by comparing the gene numbers between our initial unfiltered and final filtered transcriptomes, where gene inflation was reduced by 68% (Table 2) and our final refined transcriptome contained 36,876 genes and 76,125 isoforms.

Although not indicative of transcriptome quality, we calculated specificity, as a measure of difference between our transcriptome and other annotations, and sensitivity, which indicates how our transcriptome covers another annotation. These parameters demonstrate that our aggressive filtering does sacrifice sensitivity at the locus level by a margin of approximately 5%, and increases our specificity often by more than 10%, relative to NCBI and ENSEMBL (Additional file 5: Table S4). We have a comparable sensitivity to the Hestand transcriptome, which could be explained by adopting strict filtering approaches in both pipelines. However, the numbers of unstranded and multi-exon transcripts in the Hestand transcriptome relative to our refined version serve as the more discriminating statistics. We have approximately six fold less unstranded transcripts and more than double the multi-exon transcripts (Table 2). Regarding how our transcriptome compares to the other recent equine ISME assembly [5], which is ENSEMBL annotation guided, we have three times more matching transcripts to the ISME assembly than to the ENSEMBL annotation itself (Fig. 2a), suggesting significant improvement made by ISME annotation. However, their improvements are impaired by false inflation in the number of genes identified due to presenting most of the transcripts in two copies representing the forward and reverse strands. This inflation of ISME annotation can explain why it has different statistics from Hestand as well as our new transcriptome. Hestand et al. (2015) also observed a bias towards single exon genes, which represented approximately 55% of their whole transcriptome [7]. Our native assembly identified similar percentage of single exon transcripts, however those numbers went down to 20% after filtration of single exon pre-mRNA. We also illustrate their function by assessment of transcription in different tissues relative to the number of exons, which indicates that a majority of transcripts consisting of one or two exons occupy a large proportion of transcriptional output (Additional file 11: Figure S3). Our statistic, complex loci, also highlights a level of sensitivity as well as area for further investigation in our transcriptome. We have more than two times more complex loci, using NCBI as a reference, than Hestand. The inflated ISME complex loci numbers could be attributed to the double reporting of their transcripts. Awareness of these complex loci allows for refinement of transcriptome-wide gene structure, while a pipeline to appropriately process these loci has yet to be established.

Accurate identification of UTRs is often difficult for ab initio programs and requires sufficient support of transcription evidence. Our integrative analysis of several tissues using different library preps enabled us to achieve unpreceded extension of equine transcripts’ UTRs by an average of 3.3 kb per transcript. Indeed high coverage of CNS tissues in our analysis was an important factor,﻿ as reported previously with several transcriptomes [20–22]. Further improvements to this transcriptome would include providing tissue-specific UTR lengths and allowing for a more clear depiction of differences in gene structure between tissues. The improved UTR structure provided by our transcriptome has already shown its utility in the horse community by defining isoform and gene boundaries of *MUTYH* and *TOE1* [23] as well as providing an alternative start exon for *CYP7A1* [13].

Our final version of the transcriptome represents a collection of genes provided by eight distinct tissue-specific RNA-seq libraries. This feature allows us to extract inherent tissue-specific characteristics from the transcriptome regarding gene expression, mitochondrial gene expression and isoform usage between the tissues. With gene clustering and Panther annotation, genes corresponding and exclusive to the inherent functions of the tissue were revealed (Fig. 3a, Additional file 6: Table S5). This indicates that above any noise that was created by the different methods of cDNA library preparation or sequencing, a transcriptomic signature with biological relevance to the tissue can be deciphered. Additional to the nuclear gene expression signature, the amount of transcription occurring from the mitochondrial chromosome can stipulate how much of the sequencing resources are being allocated to mitochondrial originated genes. Across the eight tissues, one would expect the tissue with the largest numbers of mitochondria to have the largest proportion of transcriptional output allocated to mitochondrial genes, as seen with the human transcriptome [2]. Our data demonstrates these trends in the brainstem, skeletal muscle and spinal cord, however the cerebellum and retina do show an unexpectedly low mitochondrial gene load (Fig. 3c). Further research establishing the relationship between the amount of mitochondria processed in a sample for RNA-seq and the resulting mitochondrial expression loads would be beneficial to understanding how much of the transcriptional output is dominated by an individual mitochondrion. This information would also allow researchers to extract more information pertaining to mitochondria from RNA-seq data.

In addition, to gene expression patterns, isoform usage further distinguished tissue specificity. In agreement with previous retina transcriptome work [24], the retina displayed the most unique splicing with 2962 uniquely present isoforms (876 genes) and 8202 uniquely absent isoforms (5256 genes), along with a relatively high cumulative expression, highlighting its transcriptome specificity in the form of splicing. Reinforcing this specificity, Panther annotations of these unique isoforms are related to phototransduction and photoreceptor cell maintenance. The skeletal muscle was a tissue with a relatively low amount of unique tissue-specific isoforms, however, it shows utility of these isoforms with relatively high cumulative expression values (Fig. 3b) as well as comparable splicing rates (Table 3). Three tissues: retina, skin, and embryo, had shorter read lengths and were not prepared as stranded libraries and thus these data may be artificially understated in terms of transcriptome complexity.

We identified 7494 candidate novel transcripts. These novel transcripts are selected based on having no overlap with genes in current equine annotation and authenticated by their protein-coding ability and/or overlap with aligned non-horse genes or ab initio gene predictions. Our novel transcripts have a diversity of coding exons in Category I and a particular expression bias in the embryonic tissues of Category II, in which a majority of these novel transcripts contain two exons (Fig. 4c). The Category I novel transcripts highlight the deficient equine ENSEMBL annotation, the need to pool the databases to get the most transcriptome coverage and the ability of our transcriptome to capture the potentially rare novel gene models (Fig. 4a). Category II novel genes showed an expansion in two exon genes originating from the embryonic tissues, likely representing regulatory non-coding RNA, which has been found to dominate embryonic transcription in human [25, 26]. Despite the ORF requirement for Category III novel transcripts, there is an obvious enrichment for single exon transcripts and a marked reduction of total transcription level (Fig. 4c), which is indicative of non-coding RNA. Our novel gene analysis also produced a category of novel transcripts that were removed due to not having ORFs and were presumed to represent noisy transcription relating to primary transcripts, repetitive elements, sequencing errors and genome-based errors. The collection of Category III and these excluded novel transcripts may represent a repository of non-annotated non-coding RNA, which is an area that needs further annotation in the horse genome.

## Conclusions

Our transcriptome assembly pipeline not only produces flexible incorporation of additional transcriptomes, it also provides several products regarding levels of transcript filtering and appropriateness for downstream analysis. The different extractable transcriptome versions include transcriptomes after each individual filter, with the final refined transcriptome containing only genes with complete ORFs and genes aligning with other non-horse genes or ab initio gene predictions. A version of transcriptome merged with NCBI and ENSEMBL annotations achieves breadth not covered by our tissues. These transcriptomes as well as the pipeline to make each of these transcriptomes are publically available on our GitHub repository. By making the workflow public and easy to execute and manipulate, we aim to expand the spectrum of tissues embodying this transcriptome and eliminate biases in annotated genes and thus downstream differential gene analysis. As stated in our overall goals of this project, we have provided a framework for further improving the equine transcriptome and produced an equine transcriptome that expands on current equine annotations in the manner of UTR extension, isoform detection and novel gene identification.

## Methods

### RNA-seq library preparation

A total of 12 RNA-seq libraries in 8 tissues from 59 individuals (20 female, 27 males and 12 embryos) were used to prepare our transcriptome. All samples were generated by authors (Table 1) and are publically available. The brainstem, spinal cord and cerebellum were strand-specific 100 bp paired-end (PE) libraries. The skeletal muscle tissues were strand-specific PE125 bp libraries. The embryos were day 8 post ovulation and a subset of the embryo ICM (3 samples) and TE (3 samples) were unstranded PE100 bp libraries, the other subset (3 ICM and 3 TE) consisted of single end (SE) 100 bp reads. The retina RNA-seq libraries were unstranded SE80 bp libraries. The skin libraries were all unstranded and consisted of PE80 bp, SE80bp and SE95 bp reads. The brainstem, spinal cord and cerebellum RNA libraries were all rRNA-depleted, the skin, retina and skeletal muscle libraries were poly-A captured. The embryonic libraries were neither poly-A selected nor rRNA depleted, they were prepared with the Ovation® RNA-seq System V2 (NuGEN, San Carlos, CA, USA), which aims to amplify mRNA as well as non-polyadenylated transcripts. Table 1 summarizes the tissue-specific RNA-seq library parameters.

### Trimming and mapping of reads

The Illumina adaptors as well as the reads were trimmed with the sliding window quality trimmer Trimmomatic [27] with a window size of 4 and a softer quality threshold of 2 [28]. Mapping of the trimmed reads was done with Tophat2 [29] to EquCab2.0, 2007 (ftp://hgdownload.cse.ucsc.edu/goldenPath/equCab2). Cufflinks [15] was used to assemble transcripts from the aligned RNA-Seq reads. Two cerebellar samples failed assembly due to computation limitations (8 CPUs, 250 Gb RAM and 7 days) and required digital normalization [30] to 200× coverage before mapping with Tophat2.

### Filtering transcripts

Four categories of filters were used to remove likely pre-mRNA and artifactual transcriptional fragments, as summarized in Fig. 1, resulting in six versions of the transcriptome. Primary transcript filtration was done using Cuffcompare [15] between our assembly and a version of our assembly containing only multi-exon transcripts and removing transcripts overlapping with intronic regions (class codes “i”, “e” and “o”). The input trimmed RNA-seq reads were then back-mapped to the pre-mRNA free transcriptome using the quasi-mapping based software package Salmon [31]. While back-mapping, a second filtration step was implemented: low abundance transcripts in every locus were excluded with the lower threshold of a TPM (normalized read count standing for transcripts per million) less than 5% of the total TPM per locus. For the third filter, Transdecoder [32] was used to predict the ORFs and Cuffcompare [15] to determine any exonic overlap with any candidate gene locus (class codes “j”, “o”, “x” and “c”). In the Transdecoder analysis, the longest open reading frames were extracted as well as any sequences having significant homology to the Pfam and Swissprot protein databases. Finally, the removal of likely erroneous mitochondrial and short transcripts was done by a homemade script.

### Transcriptome comparisons

Comparisons of our refined transcriptome to the four public horse transcriptomes were done using Cuffcompare [15]. In any pairwise comparison, two transcripts are considered matching if they have the exact intron chain, despite differing terminal exons (class code “=”). If the transcripts do not match but share one or more splice junctions (class code “j”), these would be considered similar transcripts. A transcript is considered novel if it does not overlap with any gene model in the 2nd reference assembly (class code “u”). All other class codes including any kind of overlap with a reference annotation on the opposite strand were considered as “other”. For more detailed descriptions of the class codes provided by Cuffcompare, please see their manual [15]. Complex loci were flagged if a gene model of one assembly overlapped with 2 different gene models in the other assembly. Sensitivity and specificity relative to a given reference transcriptome were calculated per base, intron and locus for each transcriptome and reference combination as described by Burset and Guigó [14].

### Novel gene prediction

Any transcript in our final *refined* transcriptome is defined as novel if it does not overlap with a gene model in at least one of the two public equine assemblies, NCBI and ENSEMBL (Cuffcompare class code “u”). Transcripts considered novel were divided into three groups according to the degree of supportive evidence. Transcripts novel to either the NCBI or ENSEMBL assemblies with transcriptional supportive evidence from the other or any other public assembly [5, 7] were in the first category of novel transcripts. Supportive evidence is defined as any overlapping with exon sequence (Cuffcompare class code “=”,“j”,”o”,”x” or “c”). The second and third categories of novel transcripts required that the transcript be absent in all current equine transcriptomes. Transcripts in the second category have supportive evidences in non-horse alignment gene models or ab initio gene prediction tracks from the UCSC genome browser. The third category of novel transcripts included transcripts that lack such evidence but have ORFs.

### Tissue-specific characterization of the transcriptome

Tissue-specific transcriptomes were generated by back-mapping the input trimmed RNA-seq reads with Salmon [31] to the refined version of the transcriptome to obtain expression information on a tissue-specific level. A transcript is considered expressed in a given tissue if it has a TPM more than 5% of the total TPM per locus calculated from the tissue specific libraries only. The tissue specific heatmap clustered the genes using Pearson correlation and the tissues using Spearman correlation. The agglomeration method used was “average” and the distance used for the hierarchical clustering was the dissimilarity distance. Biological processes identified within the tissue-specific gene blocks were annotated with Panther [33] and reported if the *p*-values were below the Bonferroni-corrected threshold (5% experiment-wide).

### UCSC track hubs

Gene Annotation Format (GTF) files were converted into the binary bigbed files [34] using UCSC kintUtils (https://github.com/ENCODE-DCC/kentUtils). The track hub directory structure was designed as recommend by UCSC genome browser [35]. Tracks were constructed using “bigBed 12” format and multiple libraries of the same tissue were organized in composite tracks. The hub files are hosted on a github server as a part of the horse_trans repository (https://github.com/dib-lab/horse_trans).

## Additional files

Additional file 1: Table S1. Overall mapping statistics for trimmed reads with Tophat2. (DOCX 261 kb)
Additional file 2: Table S2. General statistics regarding each version of the transcriptome. (DOCX 250 kb)
Additional file 3: Table S3. Cuffcompare results between our annotation and other equine annotations. (XLSX 11156 kb)
Additional file 4: Figure S1. How our annotation compares to the NCBI database, relative to the chromosomes. Our refined version of our annotation demonstrates no bias in a certain comparisons to any particular chromosome, the yellow line represents a scale of the size of the chromosomes in megabase pair. (DOCX 155 kb)
Additional file 5: Table S4. Sensitivity (sn) and specificity (sp) analysis of all equine annotations, relative to NCBI and then ENSEMBL. Notes: *Unfiltered is the unfiltered version of our transcriptome, post filter 1 is our transcriptome after removing intronic fragments, post filter 2 is our transcriptome after removing low expressing transcripts, post filter 3 is our transcriptome after removing any transcripts without RNA-seq support from our dataset and merged trans. is our transcriptome after merging it with NCBI and ENSEMBL (DOCX 544 kb)
Additional file 6: Table S5. Panther GO biological process annotation of the embryo, skin, muscle, retina and CNS. (XLSX 60 kb)
Additional file 7: Table S6. Panther GO biological process annotation of the uniquely present transcripts in the embryo, skin, muscle, retina and CNS. (XLSX 64 kb)
Additional file 8: Table S7. Panther GO biological process annotation of the uniquely absent transcripts in the embryo, skin, muscle, retina and CNS. (XLSX 76 kb)
Additional file 9: Figure S2. Category I novel transcripts, transcripts novel to at least one equine annotation but supported by another, are most prominent in the ENSEMBL equine annotation. (DOCX 61 kb)
Additional file 10: Table S8. Statistics for annotation of novel genes with Blastp and Blastx. (DOCX 169 kb)
Additional file 11: Figure S3. Transcript expression patterns in all tissues relative to the number of exons comprising the transcript in transcripts with up to 5 exons (A) and in transcripts with over 3 exons (B). (DOCX 190 kb)

## Abbreviations

CNS: Central nervous system
ICM: Inner cell mass
RNA-seq: RNA sequencing
SNP: Single Nucleotide polymorphism
TE: Trophectoderm
UTR: Untranslated region
