# Supplementary figures and images for: Tissue resolved, gene structure refined equine transcriptome
Source: BMC Genomics. 2017 Jan 20;18:103. doi: 10.1186/s12864-016-3451-2 (PMC5251313; doi:10.1186/s12864-016-3451-2)

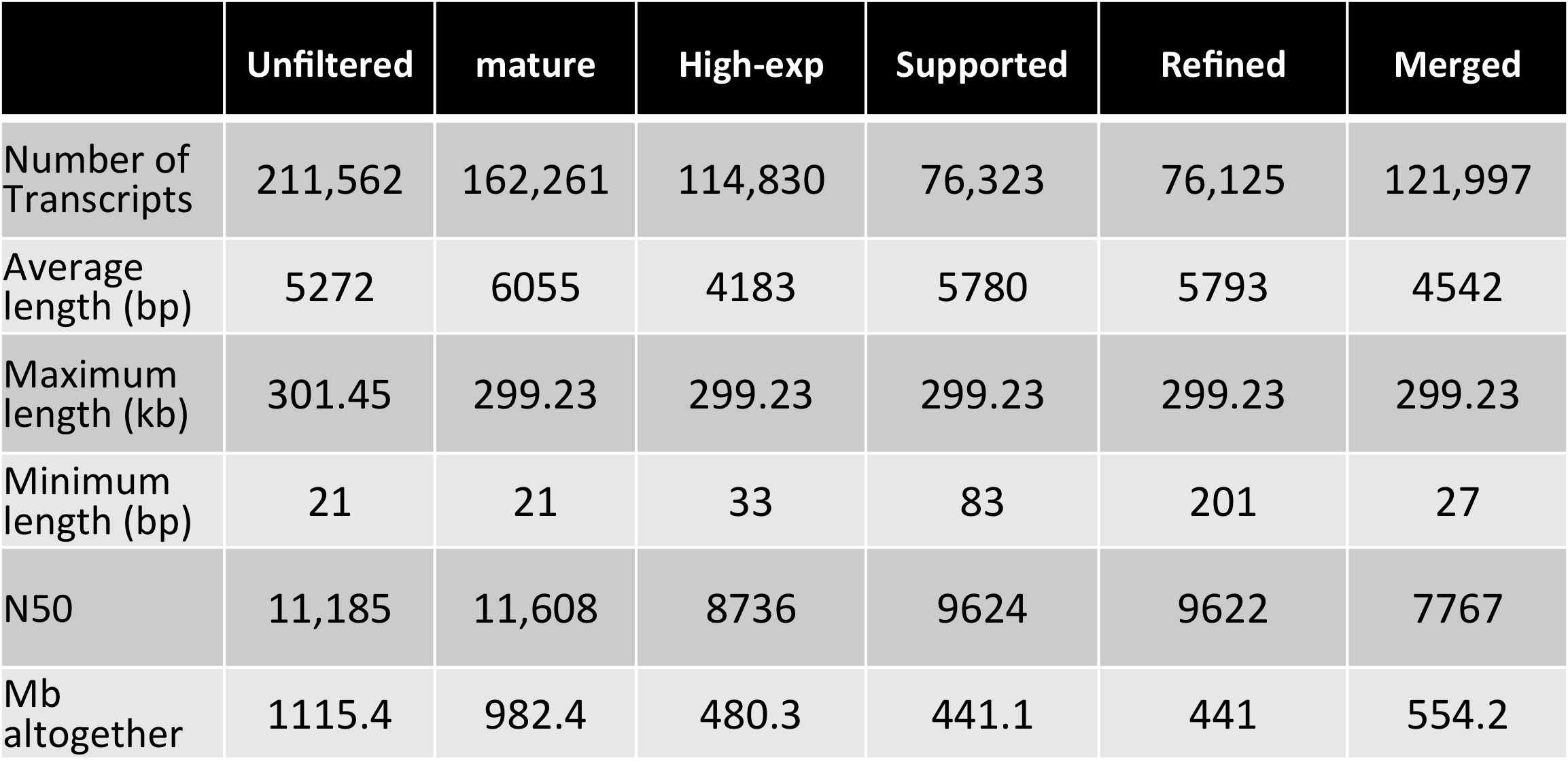

Supplement: Additional file 2: Table S2. — General statistics regarding each version of the transcriptome. (DOCX 250 kb) [file 12864_2016_3451_MOESM2_ESM.docx]

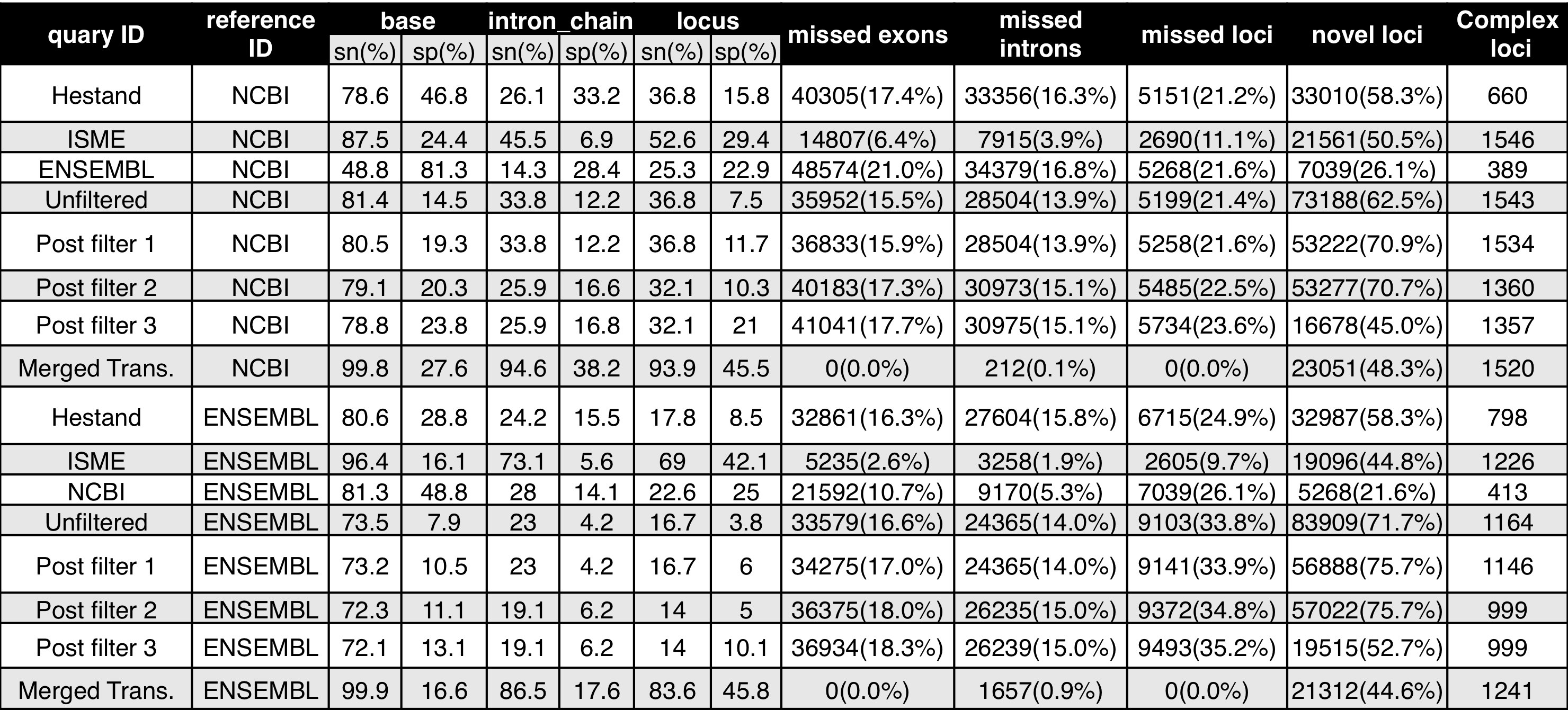

Supplement: Additional file 5: Table S4. — Sensitivity (sn) and specificity (sp) analysis of all equine annotations, relative to NCBI and then ENSEMBL. Notes: *Unfiltered is the unfiltered version of our transcriptome, post filter 1 is our transcriptome after removing intronic fragments, post filter 2 is our transcriptome after removing low expressing transcripts, post filter 3 is our transcriptome after removing any transcripts without RNA-seq support from our dataset and merged trans. is our transcriptome after merging it with NCBI and ENSEMBL (DOCX 544 kb) [file 12864_2016_3451_MOESM5_ESM.docx]

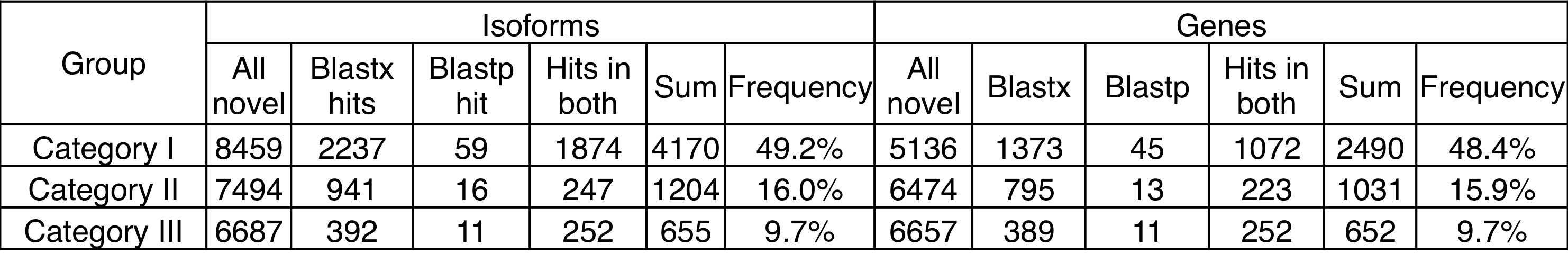

Supplement: Additional file 10: Table S8. — Statistics for annotation of novel genes with Blastp and Blastx. (DOCX 169 kb) [file 12864_2016_3451_MOESM10_ESM.docx]
