# Supplementary material for: Tissue resolved, gene structure refined equine transcriptome
Source: BMC Genomics. 2017 Jan 20;18:103. doi: 10.1186/s12864-016-3451-2 (PMC5251313; doi:10.1186/s12864-016-3451-2)
Supplement: Supplementary file 2 [file 12864_2016_3451_MOESM1_ESM.docx]

Tissue/Library	Mapping	Concordance
BrainStem/PE_100_fr.firststrand	91.11%	85.51%
Cerebellum/PE_100_fr.firststrand	85.87%	78.19%
Embryo.ICM/PE_100_fr.unstranded	50.45%	29.48%
Embryo.ICM/SE_100_fr.unstranded	78.42%	--
Embryo.TE/PE_100_fr.unstranded	51.78%	31.51%
Embryo.TE/SE_100_fr.unstranded	77.95%	--
Muscle/PE_125_fr.firststrand	92.87%	89.10%
Retina/PE_81_fr.unstranded	72.49%	68.77%
Skin/PE_81_fr.unstranded	74.42%	69.49%
Skin/SE_81_fr.unstranded	50.91%	--
Skin/SE_95_fr.unstranded	84.98%	--
SpinalCord/PE_100_fr.firststrand	91.33%	85.46%
Total	82.83%	74.99%
