# Supplementary material for: Tissue resolved, gene structure refined equine transcriptome
Source: BMC Genomics. 2017 Jan 20;18:103. doi: 10.1186/s12864-016-3451-2 (PMC5251313; doi:10.1186/s12864-016-3451-2)
Supplement: Supplementary file 3 [file 12864_2016_3451_MOESM4_ESM.docx]

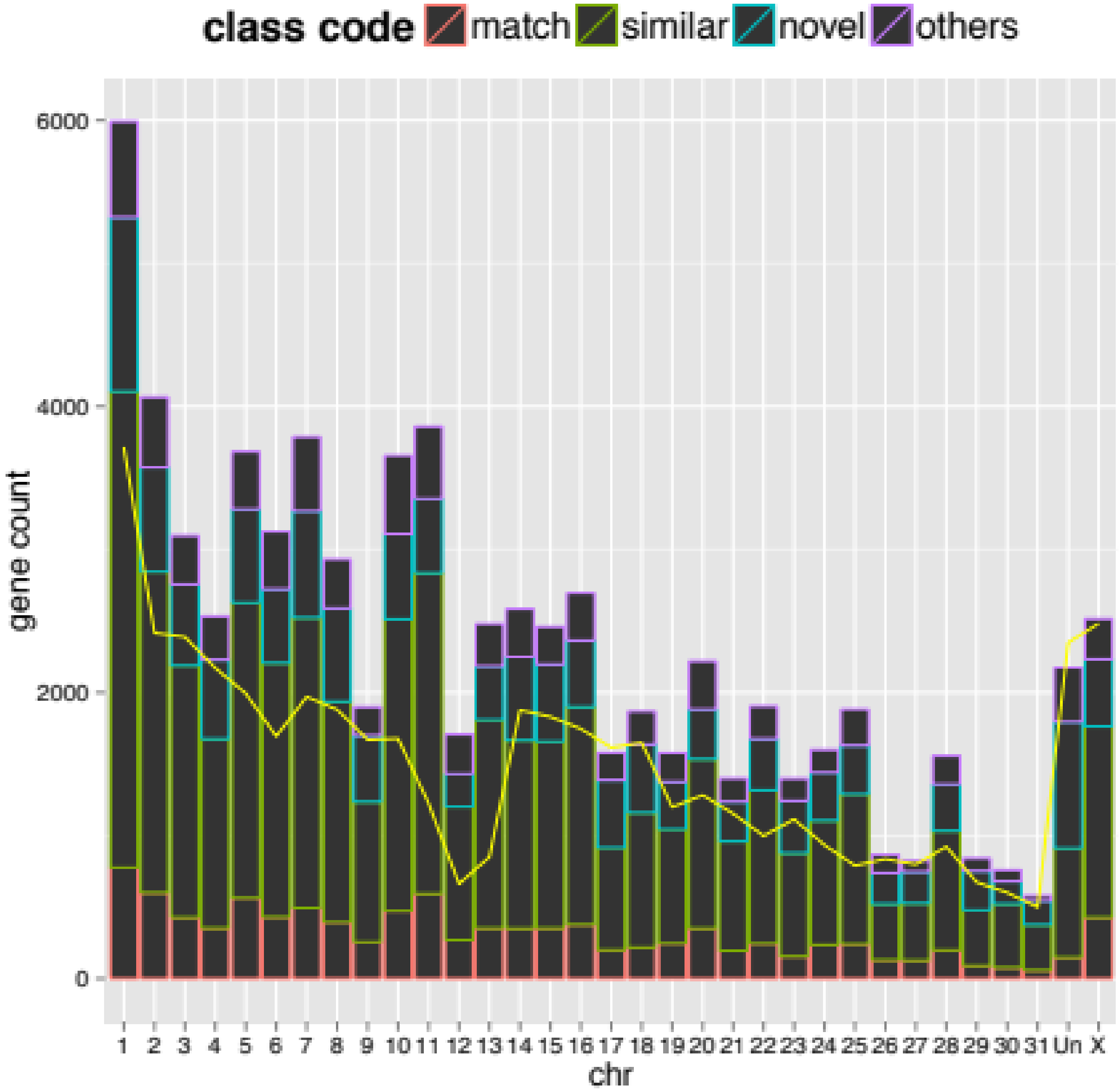

class code
match
similar
novel
others
6000
4000
2000
0
gene count
1
2
3
4
5
6
7
8
9
10
11
12
13
14
15
16
17
18
19
20
21
22
23
24
25
26
27
28
29
30
31
Un
X
chr
