# Supplementary material for: Tissue resolved, gene structure refined equine transcriptome
Source: BMC Genomics. 2017 Jan 20;18:103. doi: 10.1186/s12864-016-3451-2 (PMC5251313; doi:10.1186/s12864-016-3451-2)
Supplement: Supplementary file 4 [file 12864_2016_3451_MOESM9_ESM.docx]

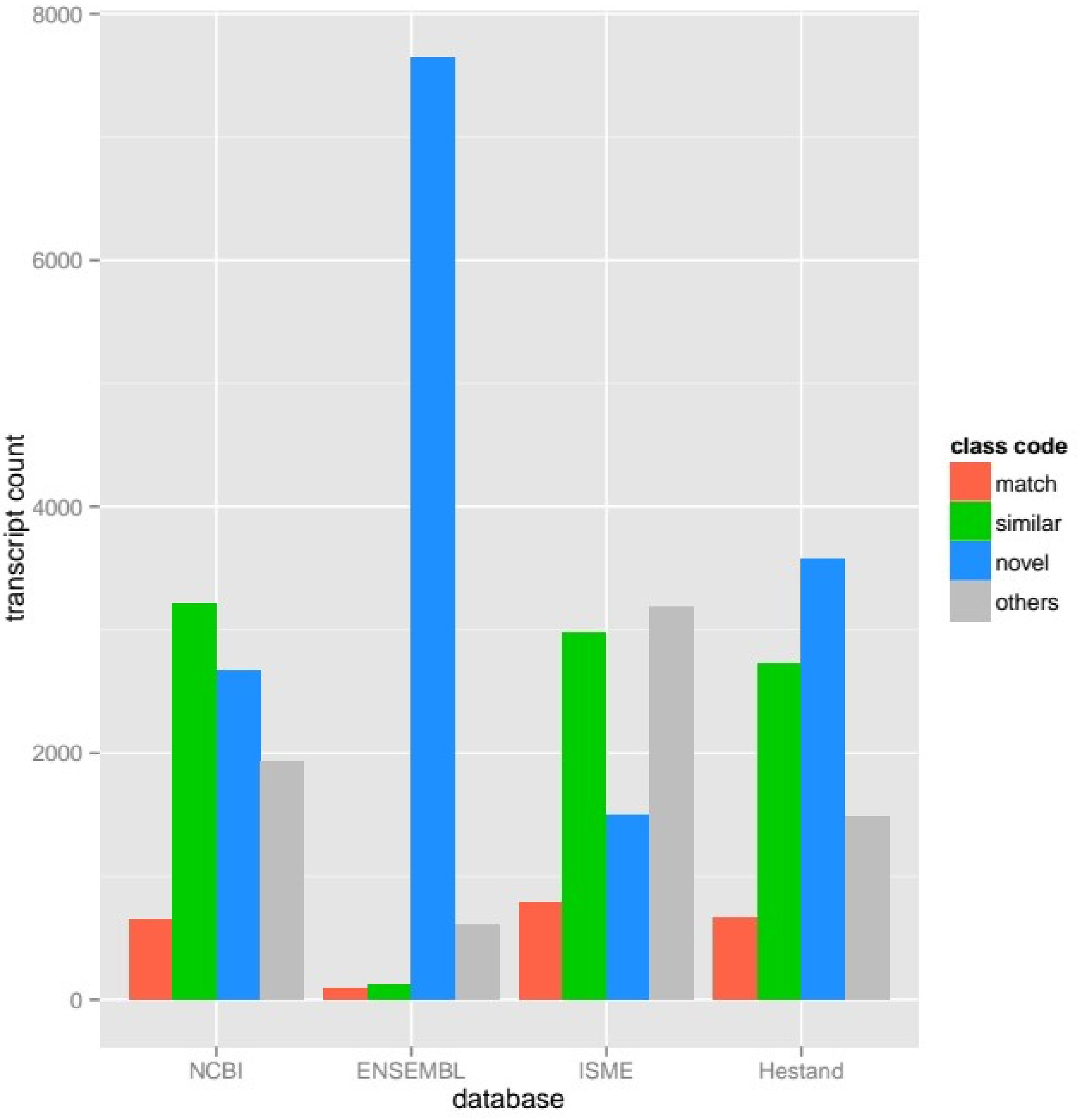

8000
6000
4000
2000
0
transcript count
NCBI
ENSEMBL
ISME
Hestand
database
class code
match
similar
novel
others
